# Spatial distribution and source apportionment of PFASs in surface sediments from five lake regions, China

**DOI:** 10.1038/srep22674

**Published:** 2016-03-07

**Authors:** Yanjie Qi, Shouliang Huo, Beidou Xi, Shibin Hu, Jingtian Zhang, Zhuoshi He

**Affiliations:** 1 State Key Laboratory of Environmental Criteria and Risk Assessment, Chinese Research Academy of Environmental Science, Beijing 100012, China; 2 College of Natural Resources and Environment, Northwest A&F University, Yangling 712100, China

## Abstract

Perfluoroalkyl substances (PFASs) have been found in environment globally. However, studies on PFAS occurrence in sediments of lakes or reservoirs remain relatively scarce. In this study, two hundred and sixty-two surface sediment samples were collected from forty-eight lakes and two reservoirs all over China. Average PFAS concentrations in surface sediments from each lake or reservoir varied from 0.086 ng/g dw to 5.79 ng/g dw with an average of 1.15 ng/g dw. Among five lake regions, average PFAS concentrations for the lakes from Eastern Plain Region were the highest. Perfluorooctanoic acid, perfluoroundecanoic acid and perfluorooctane sulfonic acid (PFOS) were the predominant PFASs in surface sediments. The significant positive correlations between PFAS concentrations and total organic carbon, total nitrogen and total phosphorus contents in sediments revealed the influences of sedimentary characteristics on PFAS occurrence. A two-dimensional hierarchical cluster analysis heat map was depicted to analyze the possible origins of sediments and individual PFAS. The food-packaging, textile, electroplating, firefighting and semiconductor industry emission sources and the precious metals and coating industry emission sources were identified as the main sources by two receptor models, with contributions of 77.7 and 22.3% to the total concentrations of C_4_-C_14_- perfluoroalkyl carboxylic acids and PFOS, respectively.

Perfluoroalkyl substances (PFASs) are a group of man-made fluorinated organic chemicals. As a result of their mass production, widespread usage and unique environmental attributes, they have spread ubiquitously in land, oceans, wildlife, human bodies, and even the remote regions such as the Arctic^1^,^2^. During production and application process, a large proportion of PFASs are released to the surface water. Perfluorooctane sulfonic acid (PFOS) and perfluorooctanoic acid (PFOA) were found as the dominant PFASs in various environmental matrices^2^. In 2009, PFOS and its salts together with perfluorooctane sulfonyl fluoride (PFOSF) were listed as the new persistent organic pollutants by the Stockholm Convention^3^. 3 M, a major manufacturer of PFOSF globally, which degrades to PFOS and related compounds, has phased out PFOSF-related products since 2000^4^. Dupont, a PFOA producer, planned to reduce the production volume by more than 85% by 2007 relative to 1999^5^. However, the production of PFOS-related chemicals in China has been rapidly increasing recently^3^,^6^. The production of polytetrafluoroethylene in China sharply increased from 6.5 kt/a in 1999 to about 64 kt/a in 2012^7^,^8^. Understanding the occurrence, spatial differentiations and potential sources of PFASs in aquatic environment on a large scale is of great importance for environmental management and criteria development for the protection of aquatic life.

Sediment, as the natural environment of benthic organisms, is one of the most important environmental sinks and reservoirs for PFASs^9^. PFASs have been found in sediments from several countries and regions, with concentrations ranging from non-detectable to several hundred nanograms per gram in dry weight (dw)^10^,^11^,^12^. The distribution pattern of PFASs reflects that PFAS concentrations are closely related to the land-use pattern, and high concentrations generally occurred in the industrialized and urbanized areas^13^. Taking the seven major river systems of China for an example, PFAS concentrations in Pearl, Yangtze and Haihe Rivers, located in the most populous and economically developed regions in China, showed higher values than those from Songhua, Liaohe, Yellow and Huaihe Rivers^2^. PFOS emissions per square kilometer every year at the provincial levels significantly increased from western to eastern China^3^. PFOS concentrations in Lake Taihu of Jiangsu Province in Eastern Plain Region, one of the most intensively industrialized provinces in China, even exceeded US EPA standards of 0.2 μg/L for PFOS in drinking water^14^,^15^. Direct discharge of untreated industiral wastewater seemed to be mainly responsible for the very high concentrations (up to 800 ng/g dw) measured in surface sediments downstream from a production base of the fluorochemical industry, and both untreated wasterwater and wastewater treatment plants (WWTPs) effluents might be also the pollution sources^11^. Industrial effluents and wastewater from WWTPs have been identified as the emission sources of PFASs leaching from PFASs-containing products^3^,^16^. Detectable concentrations of PFASs in the Arctic environment have provided a strong evidence for that atmospheric transportation and deposition of PFAS precursors can be another important source in remote areas^17^.

Studies on PFASs in aquatic environment of China have been mainly focused on the surface water, and data on PFAS concentrations in sediments are available mostly for rivers that have high concentrations in surface water. However, information on PFASs in sediments from lakes or reservoirs remains relatively scarce. This work presents the results of, to our knowledge, the first nationwide monitoring survey on surface sediments from forty-eight lakes and two reservoirs in China. In this study, seventeen PFASs have been monitored reporting their contamination status in surface sediments. Besides improving the knowledge about PFAS pollutions in Chinese sedimentary environments, spatial differentiations and the possible origins were investigated through the analysis of various methods. The specific objectives of this study were to: (1) investigate the occurrence and spatial distribution of PFASs in surface sediments from forty-eight lakes and two reservoirs in China; (2) identify the potential sources of PFASs in sediments using multivariate statistical analysis and two multivariate factor analysis receptor models; and (3) quantitatively calculate the contributions of extracted sources from the two receptor models.

## Material and Methods

### Standards and reagents

Nine stable isotope-labeled surrogate internal standards in 2 μg/mL solution mixtures, as well as a mixture of seventeen native PFASs as the external standard, were both purchased from Wellington Laboratories (Guelph, ON, Canada) (see Supplementary Table S1 for details). Perfluoro-1-[1,2,3,4,5,6,7,8^−13^C_8_]octanesulfonate (^13^C_8_-PFOS) and perfluoro-n- -[1,2,3,4,5,6,7,8^−13^C_8_]octanoic acid (^13^C_8_-PFOA) (50 μg/mL, 99%) were purchased from Cambridge Isotope Laboratories (Andover, MA, USA). All stock standards and solutions were prepared in methanol (HPLC grade, Fisher Scientific, Hampton, NH, USA) and stored in polypropylene (PP) tubes or vials at 4 °C. Ammonium acetate (NH_4_AC, HPLC grade, >97.0%), ammonium hydroxide/water (NH_4_OH/H_2_O, HPLC grade; v/v, 50%) and acetic acid (HAc, HPLC grade, >99.8%) were purchased from Alfa Aesar (Ward Hill, MA, USA). Milli-Q water was obtained from a Milli-Q Advantage A10 system (Millipore, Billerica, MA, USA) and used throughout the experiment. Solid phase extraction (SPE) columns (Oasis^®^ WAX, Weak Anion Exchange, 6 cc, 150 mg, 30 μm) were purchased from Waters Corporation (Milford, MA, USA).

### Study area and sampling

**Figure 1:**
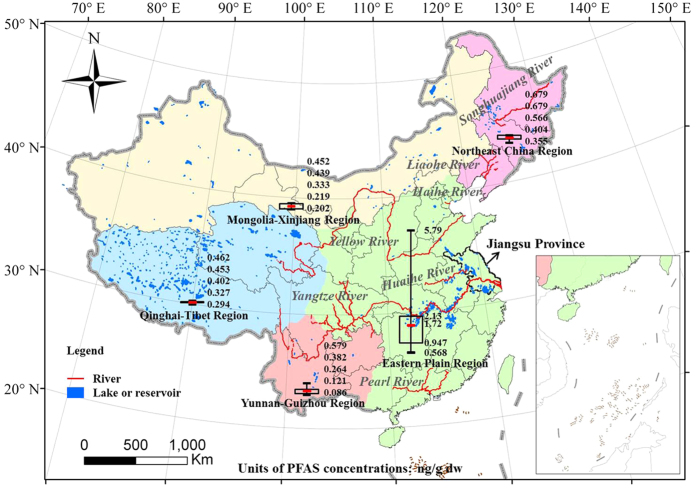
PFAS distribution in surface sediments from five lake regions across China. The top-down values in each lake region represented max, 75th, mean, 25th, min values of the ∑_12_PFASs concentrations, respectively. (We created the map using the ArcGIS 9.2 software for windows 2000/XP/VISTA/7, the SPSS 22.0 software for windows 2003/XP/VISTA/7/8 and the Microsoft Office 2010 software for windows 2003/XP/VISTA/7).

In this study, forty-eight lakes and two reservoirs were chosen from five lake regions in China, which could fully represent different geographic and limnological types of lakes in China (Fig. 1)^18^. Eastern Plain Region, located in the middle and lower reaches of the Yangtze River, middle reaches of the Huaihe, and between the Yellow River and the Yangtze River along the Yunhe River line, has the largest number of freshwater lakes^18^. The rapid development of social economy and human economic activities has great influences on both the morphology of lakes and the water quality^19^. Northeast China Region is located in a humid and sub-humid area with a continental monsoon climate. Lakes in this region are rich in organic materials and humus in sediments. Qinghai-Tibet Region regarded as “the third pole” has an average altitude above 4,000 m and is less affected by human activities^18^. There are the highest density of saline lakes and a few freshwater lakes in this region. Mongolia-Xinjiang Region is also a high density area of saline lakes with high salinity caused by the arid and semi-arid climate characteristics^20^. Yunnan-Guizhou Region, with high altitude and low latitude, is in a karst region with complicated topography. Lakes in this region are relatively small in area and rather deep, which differ from the lakes in the other lake regions. The sampling locations were selected according to their physical and biogeochemical characteristics (Wang & Dou 1998). Besides, the smaller lake area of Yunnan-Guizhou Region and Northeast China Region and the difficulties in sampling in Mongolia-Xinjiang Region and Qinghai-Tibet Region had to be taken into consideration. The geographic and limnological features of the sampling locations are shown in Supplementary Table S2 and Fig. S1.

The sampling campaign was carried out during 2010–2013. In total, two hundred and sixty-two surface sediment samples from forty-eight lakes and two reservoirs were analyzed. The sediment samples (only about 3–5 cm deep) were collected by use of a stainless grab sampler. The wet sediment samples were stored in PP bags after removing the sundries and temporarily kept in iceboxes at −4 °C. After being immediately transferred to the laboratory, the samples were stored at −20 °C. Then all sediment samples were freeze-dried at −50 °C, homogenized, sieved through a 100 mesh sieve, packed in PP bags and stored at −4 °C for further analysis.

### Sample extraction and instrumental analysis

The samples were pretreated as previously published with minor modifications and optimizations^11^. Briefly, 2 g of dry sediment samples with 1 ng of surrogate internal standards were sonicated in 15 mL of methanol at 60 °C for 30 min. After shaking for 16 h at a rate of 250 r/min f and centrifugation, the supernatant was transferred to another PP tube. The same procedures were then repeated twice. The three-part supernatants were merged, concentrated to 0.5 mL under nitrogen, diluted to 50 mL and then loaded onto an Oasis^®^ WAX single-use cartridge. The cartridges were preconditioned as reported in the previous study^11^. After evaporation the eluent to nearly dryness, all fractions were re-solubilized in 200 μL of methanol aqueous solutions (v/v, 50%) with 1 ng of ^13^C_8_-PFOA and ^13^C_8_-PFOS to further analysis. Total organic carbon (TOC) was measured by a TOC analyzer (Multi N/C 2100, Analytik Jena AG, Jena, Germany). Total nitrogen (TN) was determined using a Vario El elemental analyzer (Elementar Company, Hanau, Germany). Total phosphorus (TP) was analyzed by ammonium molybdate Spectrophotometric method^21^.

PFAS analysis was accomplished using a ultra-high performance liquid chromatography coupled to a negative electrospray ionization tandem mass spectrometer (UPLC-ESI-MS/MS, Xevo TQD, Waters Corporation, Milford, MA, USA) operated in the quantitative multiple reaction monitoring mode. PFASs were separated on an Acquity UPLC ^®^BEH C18 column (2.1 mm i.d. × 50 mm length, particle size 1.7 μm, Waters Corporation, Milford, MA, USA) with an aliquot of 10 μL injection. Detailed information on instrumental analysis has been reported in our previous study^22^.

### Quality assurance/quality control (QA/QC)

All fluorinated or glass materials were removed to avoid contaminations and sorption during sampling, extraction and analysis. Consumables used during sampling, extraction and analysis were checked with the absence of PFASs. Most of them were disposable to avoid the introduction of PFASs. All standards and chemicals (i.e. methanol, NH_4_AC, NH_4_OH/H_2_O and HAc) were examined before use, and no PFASs were found. Before extraction, solvent blanks were obtained from methanol and Milli-Q water used in every ten samples. Results showed that PFAS pollutions were absent in methanol and Milli-Q water used in each sample set. Procedural blanks were extracted by the same procedure as that followed for the sediment samples in every ten samples to ensure that PFASs were not introduced during extraction. The samples in one set would be re-extracted and reanalyzed if PFASs were detected in procedural blanks. Before sample injection, a pure solvent (methanol) was injected into the measuring system. Once PFASs were detected in the measuring system, the system would be washed until no PFASs were found.

In order to correct loses and potential matrix effects, the surrogate internal standard mixtures were added before sample extraction. Blank spike recoveries (n = 5) ranged from 78% to 112% with relative standard deviations (RSDs) <10%. Matrix spike recoveries (n = 5) were achieved with spiked samples at 2 and 20 ng/mL, and their values ranged from 69% to 115% with RSDs <15%. Recoveries of the surrogate internal standards ranged from 83% to 93%. To check for the instrumental drift, 2 ng/mL of mixed standard sample was measured with every ten samples. A pure solvent (methanol) was injected to check for carry-over between samples. Quantification was conducted using an internal standard calibration curve consisting of a concentration gradient (0, 0.02, 0.05, 0.1, 0.2, 0.5, 1, 2, 5, 10, 20, 50 ng/mL) with regression coefficients >0.99 for all PFASs. The deviation of every point from the regression line was <20% from its theoretical value. Limits of detection (LODs) and limits of quantification (LOQs) were determined from the lowest acceptable calibration standard, using a signal-to-noise ratio of 3:1 and 10:1, respectively. Detailed QA/QC measurements of PFASs are given in Supplementary Table S3.

### Data analysis

PFAS concentrations are reported on a dw basis. PFHxDA, PFOcDA, PFBS, PFHxS and PFDS were excluded in data analysis due to low detection frequencies of 7–39%. In further analysis, all concentrations lower than the LODs were reported as half of the LODs, and those lower than LOQs were reported as half of the LOQs. The total concentrations of perfluoroalkyl carboxylic acids (PFCAs) (C_4_-C_14_) and PFOS were represented by the ∑_12_PFASs. Data for hierarchical cluster analysis (HCA) were further log-normalized and then Z-transformed by taking the raw scores, subtracting their means, and dividing by their standard deviations. HCA was conducted to screen abnormal samples from dataset using Ward’s method and block as a measure of similarity. Pearson correlation test (2-tailed) was used to examine the possible relationships among PFASs, gross domestic product (GDP) and the total sewage amount of the provinces and municipalities, and various sediment parameters at a significant level of 0.01, which obeyed lognormal distributions or normal distributions. Data analyses were carried out with MassLynx V4.1 (Waters Corporation, Milford, MA, USA), Office 2010 (Microsoft Incorporated, Redmond, WA, USA), SPSS 22.0 (SPSS Incorporated, Chicago, IL, USA), Matlab R2014b (MathWorks Incorporated, Natick, MA, USA), ArcGIS 9.2 (ESRI, Redlands, CA, USA) and US EPA PMF 5.0.

### Receptor models

The principal component analysis-multiple linear regression (PCA-MLR) and positive matrix factorization (PMF) models were employed to apportion sources and calculate the contributions of extracted sources. These two receptor models do not need source profiles and are used under some assumptions^23^. Due to high energy of carbon-fluorine bonds, all PFASs studied in this work are strongly stable^24^,^25^. Thus, it was assumed that PFAS species studied did not change as they moved from source to receptor and did not react with each other, as described in the previous study, and so forth^23^. Generally, they can be described by the following equation (1):


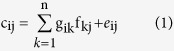


where 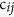 is the concentration of *i*th species for the *j*th sample; 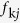 is the contribution of the *k*th source to the *j*th sample; *g*_*ik*_ is the *i*th species concentration from the *k*th source; and *e*_*ij*_ is the error^26^. Details on the present application of these two receptor models are described in the following sub-chapters.

#### PCA-MLR model

Assuming a linear relationship between the total mass concentrations and the contributions of each species, PCA-MLR was applied to the dataset using SPSS 22.0 and Matlab R2014b. Prior to data analysis, all data were transformed into a dimensionless standardized form as the equation (2). Only factors with eigenvalues higher than one were evaluated, and the variables were considered to identify source categories if their factor loadings were >0.600 (absolute value). The model was carried out using Varimax rotation with Kaiser normalization. The rotated principal component (PC) scores were computed using the rotated PC coefficient matrix and the Z-score matrix. The rotated ‘absolute zero’ PC scores were calculated using the rotated PC coefficient matrix and the ‘absolute zero’ Z-score matrix. The absolute PC scores (APCS) for each component was obtained by subtracting the ‘absolute zero’ PC scores from the rotated PC scores. The regression between the Z-scores and the APCS were then conducted using Matlab R2014b. The contributions of different sources to the ∑_12_PFASs could thereby be obtained. More detailed description of the PCA-MLR model could be found in the study of Thurston and Spengler^27^.


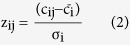


where *z*_*ij*_ is the standardized concentration of *i*th species for the *j*th sample and often termed the Z-score; 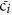 is the average concentration of *i*th species over all sampling sites; and σ_*i*_ is the standard deviation of the concentration of *i*th species.

#### PMF model

The major differences of the PMF model from the PCA-MLR model are that this model runs with non-negative constraints on variables and considers the uncertainty for each variable. Besides, the factors in PMF are not necessarily orthogonal to each other. The matrix of measured uncertainties was calculated by the LOD for each species and the error. If the concentration ≤the LOD, uncertainty = 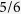 × LOD^28^, otherwise, uncertainty = 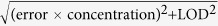
29. Roughly based on the RSDs, the errors of all species were set to 0.05. The extra modeling uncertainty was set to a value of 5%.

The robust Q was calculated by excluding outliers, defined as samples for which the uncertainty-scaled residual is greater than four, while the true Q is calculated including all points. Different runs were carried out to improve results by down weighting the species with low S/N from “strong” to “weak”. In this way, twelve variables were all included by considering them “strong”. The ∑_12_PFASs as the total variable was considered as “weak”. The factor number (P) was gradually adapted from 2 to 4. Increasing P is supported only if the decrease of Q is significant. Consequently, the PMF model was run with three factors. All runs converged to find a similar global minimum. The robust Q was 30,363 and the true Q was 51,356. After a reasonable solution was obtained, the uncertainties in the modelled solution were further estimated by performing a total of 300 bootstrap runs^30^. All runs converged with a minimum r-value of 0.6 for base-boot factor mapping. Residuals were checked to be between −3 and 3 for all species and at least 96% of the observations. Fpeak = 0.5 was adopted in the model fitting.

## Results and Discussion

### Spatial distribution of PFASs in sediments

The spatial distribution patterns of PFAS concentrations in surface sediments from five lake regions of China are depicted in Fig. 1. The descriptive statistics of PFAS concentrations in surface sediments from five lake regions are summarized in Table 1. Quantifiable concentrations of PFASs in surface sediments were all found in forty-eight lakes and two reservoirs, ranging from 0.086 ng/g dw to 5.79 ng/g dw with an average of 1.15 ng/g dw. The average PFAS concentrations in surface sediments from Eastern Plain Region had an extreme value of 1.72 ng/g dw and was at least 3.0 times higher than any of the other four lake regions. It is well-known that Eastern Plain Region, as the congeries of towns and population, has brought together several major industrial and agricultural districts in China and suffered the most intense human interference^18^. The average PFAS concentrations in surface sediments had a significant increase from Yunnan-Guizhou Region in the upper reaches of the Yangtze River to Eastern Plain Region in the lower reaches of the Yangtze River, untouched by the dilution effect of the water current. It suggested that the possibility of terrestrial discharges of PFASs existed along the Yangtze River^11^. The highest PFAS levels in surface sediments from Eastern Plain Region could be largely attributed to human contamination from autochthonous sources such as heavy industries^31^, urban activities^32^, airports^12^, and agricultural activities^33^. PFASs have been found in surface sediments from the remote Qinghai-Tibet Region and Mongolia-Xinjiang Region. It most likely resulted from the transportation of volatile PFAS precursors via the atmosphere, degradation by atmospheric oxidation to PFASs and subsequent dry and wet deposition^34^,^35^.

The average PFAS concentrations in surface sediments from the lakes in Jiangsu Province were extremely higher than those in other provinces. It could result from the fast growing fluorochemical industries in Jiangsu Province, as PFASs-related industries are the significant sources of PFAS releases to the environment in China^2^,^31^. Results of Pearson correlation analysis (*r* = 0.825–0.981, *p* = 0.001–0.003) showed that C_8_ and C_10_-C_14_-PFCAs, C_8_-perfluoroalkane sulfonic acids (PFSAs) and the ∑_12_PFASs concentrations had a significant positive correlation with GDP and the total sewage amount of the provinces and municipalities in 2013, consistent with the previous study^32^,^36^. Thus, it could be concluded that PFAS emissions were closely related to the regional urbanization and industrialization^2^. Human activities, such as discharges of industrial wastewater and domestic sewage as well as diffusions of polluted urban and agricultural runoff, played an important role in PFAS occurrence^37^,^38^,^39^.

Geographical variations can be distinguished from the comparisons of PFASs in surface sediments from different regions (see Supplementary Fig. S2). In general, PFAS concentrations in surface sediments from China reached the low end of the worldwide levels, with the exception of those from Jiangsu Province, which was found to have the highest concentrations among the Chinese provinces investigated (up to 2.45 ng/g dw).

### Detection frequency, concentrations and compositions of PFASs

Seventeen target PFASs were all detectable in this study, as displayed in Table 1. Twelve PFASs, including C_4_-C_14_-PFCAs and PFOS, were quantified in surface sediments from at least 80% of lakes and reservoirs. Among them, C_5_, C_8_-C_9_ and C_11_-PFCAs spread over the surface sediments from forty-eight lakes and two reservoirs. PFOA, PFUnDA and PFOS were found to be the predominant PFASs, with averages of 0.221, 0.200 and 0.198 ng/g dw, accounting for about 19.2%, 17.4% and 17.2% relative to the ∑_12_PFASs, respectively. Contributions of the long-chained PFCAs (C_9_-C_14_) were about 2.8 times higher than those of the short-chained PFCAs (C_4_-C_7_) to the ∑_12_PFASs. This characteristic came to light especially in surface sediments from the lakes in Eastern Plain Region. The long-chained PFCAs (C_9_-C_14_) contributed 50.7% of the ∑_12_PFASs, whereas contributions of the short-chained PFCAs (C_4_-C_7_) were only 13.5%. The long-chained PFCAs (C_9_-C_14_), along with PFOA, were also the major PFASs in surface sediments from the lakes and reservoirs in Northeast China Region. However, the short-chained PFCAs (C_4_-C_7_) accounted for the major portion of the ∑_12_PFASs in surface sediments from the lakes in Mongolia-Xinjiang Region and Qinghai-Tibet Region, both accounting for 58.4% of the ∑_12_PFASs, respectively. Consistent with this, contributions of the short-chained PFCAs (C_4_-C_7_) were slightly higher than those of the long-chained PFCAs (C_9_-C_14_) in surface sediment from Yunnan-Guizhou Region, and PFOA accounted for a large proportion of the ∑_12_PFASs, a total of 41.4%.

In most previous studies (see Supplementary Fig. S1), PFOS, PFOA and the long-chained PFCAs were the predominant compounds detected in surface sediments from the lakes and harbors. PFAS concentrations in surface sediments from forty-eight lakes and two reservoirs in China showed the similar characteristics, represented by the lakes from Eastern Plain Region. Unlike them, PFOS, PFOA and the short-chained PFCAs (C_4_-C_7_) were predominant in surface sediments from three lakes in the Canadian Arctic^17^, and Lake Constance and alpine lakes in Austria^40^. It was similar to PFAS concentrations reported for Qinghai-Tibet Region and Mongolia-Xinjiang Region of China in this study.

### Influence of sedimentary characteristics on PFAS occurrence

The relationships between PFAS concentrations and TOC contents in surface sediment from five lake regions were studied. Preferable regularity only happened to the surface sediments from Eastern Plain Region. A significant positive correlation was found between the ∑_12_PFASs concentrations and TOC contents (*r* = 0.765, *p* = 0.000) without considering the singular values. The regression for the ∑PFCAs (C_4_-C_14_) (*r* = 0.797, *p* = 0.000) showed more significant relationships than that for PFOS (*r* = 0.537, *p* = 0.000). Concentrations of the long-chained ∑PFCAs (C_8_-C_14_) (*r* = 0.751, *p* = 0.000) showed a stronger correlation with TOC contents than those of the short-chained ∑PFCAs (C_4_-C_7_) (*r* = 0.334, *p* = 0.001). For individual PFAS, PFDA concentrations showed a strong correlation with TOC contents (*r* = 0.727, *p* = 0.000). As for all studied provinces, concentrations of C_8_-C_10_, C_13_-PFCAs, the ∑PFCAs (C_4_-C_14_), the ∑PFCAs (C_8_-C_14_), the ∑_12_PFASs and PFOS were all significantly positive correlated with TOC contents in surface sediments from Jiangsu Province (*r* = 0.786–0.884, *p* = 0.000). Organic content was recognized as the dominant influence factor on adsorption of anionic PFAS surfactants, suggesting the importance of hydrophobic interactions^41^,^42^,^43^. The increase of perfluorocarbon chain length would strength the adsorption of PFASs on sediments^43^. There were also significantly positive correlations between C_5_-C_10_ and C_12_-C_14_-PFCAs and TP, PFOS and TP, and the ∑_12_PFASs and TN in surface sediments from Jiangsu Province (*r* = 0.778–0.833, *p* = 0.000). Interactions between other persistent organic pollutants (i.e. polychlorinated biphenyls and Di-2-ethylhexyl phthalate) and eutrophication were also found^44^,^45^, which might be achieved mainly by acting on the biomass in aquatic ecosystems^46^. In total, adsorption of PFASs on sediments was affected by not only organic matters, but also inorganic materials, indicating that both hydrophobic and electrostatic effects played a role in the transport and fate of PFASs in sediments.

The relationships between PFAS concentrations and TOC contents showed obvious regional differences among five lake regions, which might be attributed to human disturbances in varying degrees^47^. At first, it should be noted that PFASs do not exist naturally in environment, and PFAS pollutions largely originated from their own manufacture and use and discharged into the aquatic systems through landfill leachates^16^, urban runoff^48^, and sewage discharge^49^. Among five lake regions, Eastern Plain Region was the most severely affected by human activities, especially that this region had an important status on the industrial development in China^19^. The positive correlations between PFAS concentrations and TOC contents in Eastern Plain Region might be attributed to their co-emissions^50^. The irrelevance between PFAS concentrations and TOC contents in these four lake regions might be ascribed to the absence of co-emissions between PFASs and TOC.

### Hierarchical cluster analysis and correlation analysis

**Figure 2:**
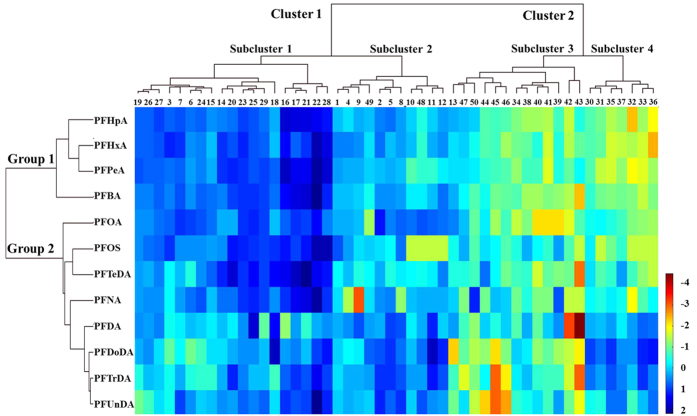
Results of a two-dimensional HCA heat map for forty-eight lakes and two reservoirs (top of the map) and twelve PFASs (right of the map). Lakes and reservoirs corresponding to serial numbers of 1–50 are displayed in Supplementary Table S2. (We created the map using the Matlab R2014b software for windows 2000/2003/XP/VISTA/7/8).

Before source apportionment by receptor models, HCA was employed to preliminarily investigate the possible origins of PFASs. Both clusters were distinguished for sampling sites and PFASs (Fig. 2). Sampling sites with similar source categories would be grouped into one cluster. Two well-differentiated clusters were observed in surface sediments from forty-eight lakes and two reservoirs. Cluster 1 consisted of all lakes (excluding Lake Nvshan) from Eastern Plain Region, and Cluster 2 consisted of all lakes (excluding Lake Xingkai and Dahuofang Reservoir) from the other four lake regions. Subcluster 1 in Cluster 1 and Subcluster 4 in Cluster 2 contained all lakes from Jiangsu Province in Eastern Plain Region and Yunnan-Guizhou Region, respectively. Subcluster 2 in Cluster 1 and Subcluster 3 in Cluster 2 included most of the lakes from Anhui Province in Eastern Plain Region and the other 2 plateau regions, respectively. Three lakes and one reservoir in Northeast China Region were separately grouped into Subcluster 2 in Cluster 1 and Subcluster 4 in Cluster 2, suggesting complicated source categories. As for PFASs, PFOS, PFOA and the long-chained PFCAs (C_9_-C_14_) were included in one group, while the short-chained PFCAs (C_4_-C_7_) were clustered into the other group. The compounds in one group might come from the same or similar pollution sources^51^. According to the results from Fig. 2, PFASs in surface sediments from forty-eight lakes and two reservoirs might originate from two sources.

Besides HCA, Pearson correlation test among various PFASs in surface sediments was performed based on the lognormal or approximately lognormal data (Table 2). Consistent with the result of HCA, significant positive correlations (*r* = 0.701–0.942, *p* = 0.000, in boldface) were observed among PFOS, C_8_-C_14_-PFCAs and the ∑_12_PFASs, suggesting that they might derive from the same or similar sources. As the short-chained PFCAs, there were also positive correlations between PFPeA and PFHxA, PFHxA and PFHpA, with r of 0.800 and 0.782 (*p* = 0.000), respectively.

### Source apportionment

#### Source apportionment by the PCA-MLR model

The PFAS concentration datasets were analyzed further to quantitatively calculate the contributions of possible sources to the ∑_12_PFASs in surface sediments by use of the PCA-MLR and PMF models. The rotated factor loadings in the PCA-MLR analysis are displayed in Table 3, which shows that the first factor (55.41% of the total variance) had high loadings for PFOS and C_7_-C_14_-PFCAs. PFOA have been widely used in paper food packaging^52^,^53^,^54^. At least 92.5% of cumulative source-specific environmental releases of PFOA and its salts in China were estimated directly from PFCA manufacture, industrial and consumer uses for the period 2004–2012^55^. Manufacture and industrial uses, including textile treatment, metal plating, firefighting and semiconductors, were also identified as the crucial sources of PFOS emissions in China^3^. Thus, factor 1 can be identified as the food-packaging, textile, electroplating, firefighting and semiconductor industry emission sources. The second factor (15.60% of the total variance) got high loadings for C_4_-C_6_-PFCAs. The short-chained PFCAs can be considered as the alternatives of PFOS, PFOA and related compounds or the degradation products of their corresponding precursors^6^. It indicated that factor 2 can be associated with the on-going transition to shorter perfluoroalkyl chain compounds. C_4_-C_6_-PFCAs were mainly used as synthesis precious metals flotation agent and stain- and grease-proof coatings^10^. Therefore, factor 2 can be identified as the precious metals and coating industry emission sources.

The contributions of two factors obtained from the PCA analysis was determined by the MLR analysis. The MLR analysis yielded excellent coefficients for two factor scores at a stipulated minimum 95% confidence. The APCS matrix for PFAS contributions of two factors obtained from the PCA-MLR model were expressed using the following equation (3):


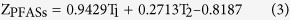


The coefficients were set as t, and the contributions of each PC were calculated using the following equation (4):


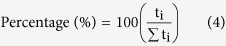


The results showed that the first factor contributed 77.7% to the ∑_12_PFASs. The second factor contributed approximately 22.3% to the ∑_12_PFASs.

#### Source apportionment by the PMF model

A total of three sources were chosen as the optimal number for the PMF model (Table 3). Unlike PCA, a high value for the source factors in the PMF model does not necessarily show that the variable is highly correlated with a source^56^. The first source (43.2% of the sources) was dominated by C_8_-C_13_-PFCAs, which was similar to factor 1 in the PCA-MLR analysis. Source 1 could be identified as the food-packaging industry emission sources. The second source (33.6% of the sources) was predominately loaded with a high concentration of PFOS and slightly weighted by PFTeDA, which were both classified into factor 1 in the PCA-MLR analysis. Source 2 could be associated with the textile, electroplating, firefighting and semiconductor industry emission sources. The third source had the smallest PFAS contribution (23.2%) and was heavily loaded with C_4_-C_7_-PFCAs. Source 3 was identified as the precious metals and coating industry emission sources.

#### Comparison of PCA-MLR and PMF results

To better understand PFAS sources, results obtained from these two receptor models were compared and estimated. When comparing these two models, it is proposed that the following four conditions must be fully considered: the fitting degree between the predicted and measured ∑_12_PFASs concentrations in a certain model, the fitting degree between the predicted ∑_12_PFASs concentrations among different models, the numbers and characteristics of identified sources as well as the contributions of each extracted source to the ∑_12_PFASs.

**Figure 3:**
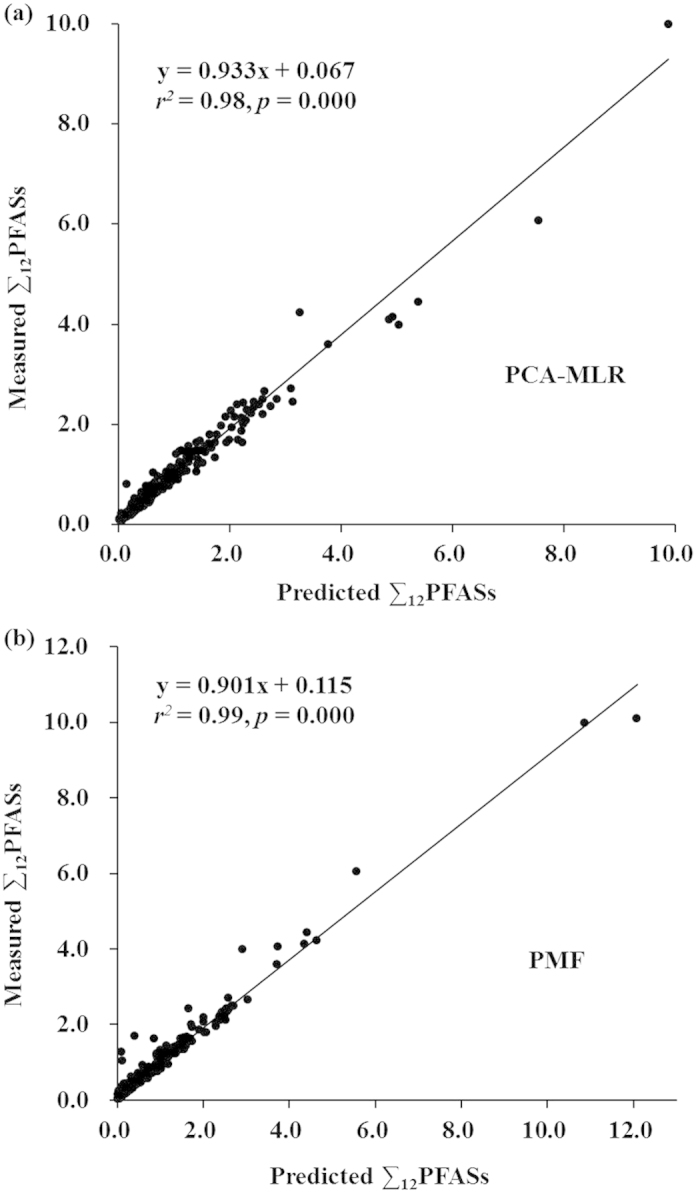
Fitting plots between the modeled and measured ∑_12_PFASs concentrations (ng/g dw) for all the surface sediments: (**a**) PCA-MLR, (**b**) PMF. (We created the map using the Microsoft Office 2010 software for windows 2003/XP/VISTA/7).

An intra-comparison was conducted by performing linear regression between the measured concentrations of PFAS species against those predicted by a certain model. As exhibited in Fig. 3, the PCA-MLR and PMF models both provided good correlations between the predicted and measured PFAS concentrations with r^2^ values of 0.98 and 0.99 (*p* = 0.000), respectively. Very similar slopes were detected in both cases and the intercepts did not vary greatly. Thus, these two models could track the ∑_12_PFASs as well as the mass explained in surface sediments. The correlation coefficients as well as the error percentages between the predicted and measured concentrations of individual PFAS species were also used to evaluate these two models’ performance (Table 4). Generally, the r^2^ were high, suggesting that these two models reproduced well the spatial evolution of individual PFAS species. As for the errors, the PCA-MLR model consistently produced almost the identical predicted values of individual PFAS concentrations as the measured ones. The PMF model generated consistently the predicted values less than the measured ones, with the absolute errors in the range of 0–33%, and there are exceptions to PFOS, with the positive error of 15%.

**Figure 4:**
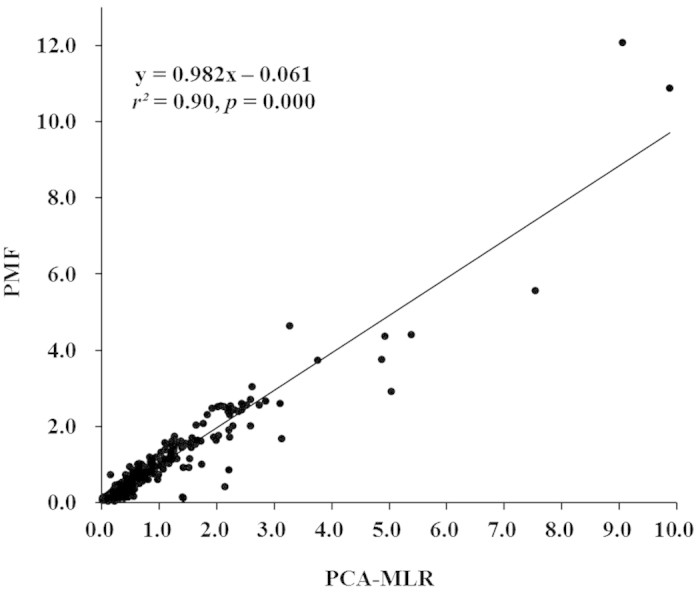
Fitting plot between the predicted ∑_12_PFASs concentrations (ng/g dw) from the PCA-MLR model and the PMF model. (We created the map using the Microsoft Office 2010 software for windows 2003/XP/VISTA/7).

Good correlations (*r*^*2*^ = 0.90, *p* = 0.000) were also observed well in an inter-comparison of the predicted ∑_12_PFASs concentrations of the PCA-MLR and PMF models (Fig. 4). The regression between the PCA-MLR and PMF models displayed a good slope very close to unity, with a small intercept of −0.061. In regard to the experimental evidence on source apportionment, two and three sources were identified by the PCA-MLR and PMF models, respectively, indicating that PMF model could track more specifically PFAS sources (Table 3). Though there were differences in the numbers of identified sources from these two models, PFAS species in factor 1 in the PCA-MLR analysis have included all PFAS species in sources 1 and 2 in the PMF analysis. The contributions of factor 1 in the PCA-MLR analysis to the ∑_12_PFASs were approximately equal to the sum of those of sources 2 and 3 in the PMF analysis. The food-packaging, textile, electroplating, firefighting and semiconductor industry emission sources and the precious metals and coating industry emission sources were identified as the two main sources, with contributions of 77.7% and 22.3% to the ∑_12_PFASs, respectively. In further studies, it is essential to develop multiple source apportionment techniques to identify the potential source categories and quantify the source contributions.

## Additional Information

**How to cite this article**: Qi, Y. *et al.* Spatial distribution and source apportionment of PFASs in surface sediments from five lake regions, China. *Sci. Rep.* **6**, 22674; doi: 10.1038/srep22674 (2016).

## Supplementary Material

Supplementary Information

## Figures and Tables

**Table 1:** Summary statistics of PFAS concentrations (ng/g dw) in surface sediments.

	Analytes	Eastern Plain Region (n = 29)^a^				Mongolia-Xinjiang Plateau (n = 5)				Qinghai-Tibet Plateau (n = 4)				Yunnan-Guizhou Plateau (n = 8)				Northeast China Region (n = 4)			
		Min^b^	Max^c^	Mean	DF (%)^d^	Min.	Max.	Mean	DF (%)	Min.	Max.	Mean	DF (%)	Min.	Max.	Mean	DF (%)	Min.	Max.	Mean	DF (%)
PFCAs	PFBA	0.017	0.305	0.090	100	0.027	0.118	0.073	100	0.050	0.113	0.080	100	0.001	0.090	0.041	88	0.018	0.084	0.050	100
	PFPeA	0.005	0.141	0.048	100	0.032	0.082	0.056	100	0.026	0.090	0.060	100	0.005	0.015	0.010	100	0.010	0.058	0.034	100
	PFHxA	0.011	0.173	0.055	100	0.019	0.106	0.052	100	0.029	0.081	0.053	100	0.001	0.017	0.007	75	0.003	0.041	0.022	100
	PFHpA	0.007	0.190	0.040	100	0.010	0.022	0.014	100	0.013	0.042	0.034	100	0.001	0.030	0.009	88	0.004	0.050	0.023	100
	PFOA	0.006	1.10	0.296	100	0.020	0.105	0.066	100	0.084	0.114	0.099	100	0.028	0.365	0.109	100	0.044	0.485	0.214	100
	PFNA	0.056	0.296	0.138	100	0.007	0.036	0.019	100	0.009	0.026	0.016	100	0.003	0.038	0.012	100	0.009	0.165	0.061	100
	PFDA	0.030	1.46	0.206	100	0.003	0.015	0.008	100	0.004	0.007	0.006	100	0.001	0.020	0.007	88	0.010	0.026	0.020	100
	PFUnDA	0.074	1.22	0.326	100	0.002	0.021	0.013	100	0.004	0.012	0.009	100	0.007	0.035	0.020	100	0.036	0.093	0.075	100
	PFDoDA	0.004	0.357	0.071	100	0.001	0.004	0.002	80	0.001	0.005	0.002	100	0.001	0.009	0.005	100	0.005	0.014	0.010	100
	PFTrDA	0.026	0.520	0.111	100	0.003	0.009	0.006	100	0.001	0.007	0.003	75	0.003	0.013	0.008	100	0.011	0.029	0.021	100
	PFTeDA	0.001	0.076	0.018	90	0.001	0.004	0.002	40	0.001	0.007	0.003	75	0.001	0.008	0.003	88	0.001	0.007	0.003	75
PFSAs	PFOS	0.027	1.80	0.319	100	0.008	0.051	0.024	100	0.005	0.101	0.038	100	0.001	0.165	0.032	88	0.020	0.042	0.031	100
∑_12_PFASs		0.568	5.79	1.72	100	0.202	0.452	0.333	100	0.294	0.462	0.402	100	0.086	0.579	0.264	100	0.355	0.679	0.565	100

^a^n indicates the number of lakes and reservoirs analyzed.

^b^Min. means minimum.

^c^Max. means maximum.

^d^DF means detection frequency.

**Table 2:** Pearson correlations among PFASs.

Analytes	PFBA	PFPeA	PFHxA	PFHpA	PFOA	PFNA	PFDA	PFUnDA	PFDoDA	PFTrDA	PFTeDA	PFOS
PFPeA	0.562^**^											
PFHxA	0.561^**^	0.800^**^										
PFHpA	0.349^*^	0.646^**^	0.782^**^									
PFOA	0.205	0.239	0.296^*^	0.389^**^								
PFNA	0.291^*^	0.338^*^	0.435^**^	0.502^**^	0.513^**^							
PFDA	0.357^*^	0.355^*^	0.491^**^	0.570^**^	0.650^**^	0.850^**^						
PFUnDA	0.146	0.159	0.286^*^	0.446^**^	0.658^**^	0.817^**^	0.906^**^					
PFDoDA	0.062	0.114	0.230	0.435^**^	0.627^**^	0.701^**^	0.875^**^	0.937^**^				
PFTrDA	0.169	0.159	0.314^*^	0.468^**^	0.554^**^	0.781^**^	0.890^**^	0.942^**^	0.926^**^			
PFTeDA	0.177	0.058	0.194	0.379^**^	0.505^**^	0.468^**^	0.749^**^	0.735^**^	0.858^**^	0.770^**^		
PFOS	0.350^*^	0.399^**^	0.485^**^	0.462^**^	0.626^**^	0.659^**^	0.874^**^	0.723^**^	0.744^**^	0.710^**^	0.720^**^	
∑_12_PFASs	0.414^**^	0.460^**^	0.580^**^	0.637^**^	0.785^**^	0.794^**^	0.930^**^	0.871^**^	0.838^**^	0.817^**^	0.702^**^	0.858^**^

Spearman rank correlations among PFASs.

^**^Correlation is significant at the 0.01 level (2-tailed).

^*^Correlation is significant at the 0.05 level (2-tailed).

**Table 3:** Source profiles of PFASs obtained from the PCA-MLR and PMF models.

Compound	PCA factor loadings		PMF source composition (ng/g dw)		
	Factor 1	Factor 2	Source 1	Source 2	Source 3
PFBA	−0.015	**0.719**	0.0034	0.0000	**0.0509**
PFPeA	0.027	**0.900**	0.0000	0.0034	**0.0316**
PFHxA	0.366	**0.766**	0.0000	0.0045	**0.0317**
PFHpA	**0.730**	0.461	0.0058	0.0034	**0.0122**
PFOA	**0.823**	0.202	0.0756	0.0454	0.0471
PFNA	**0.698**	0.247	0.0570	0.0022	0.0093
PFDA	**0.862**	0.154	0.0468	0.0290	0.0038
PFUnDA	**0.900**	0.034	0.1124	0.0197	0.0067
PFDoDA	**0.951**	0.068	0.0140	0.0116	0.0002
PFTrDA	**0.903**	0.100	0.0332	0.0097	0.0021
PFTeDA	**0.834**	0.015	0.0033	0.0034	0.0000
PFOS	**0.674**	0.028	0.0224	0.1602	0.0072
Possible source	Food-packaging, textile, electroplating, firefighting and semiconductor industry	Precious metals and coating industry	Food-packaging industry	Textile, electroplating, firefighting and semiconductor industry	Precious metals and coating industry
Contribution (%)	**77.7**	**22.3**	**43.2**	**33.6**	**23.2**

**Table 4:** Information of individual PFAS concentrations (ng/g dw) obtained by the PCA-MLR and PMF models.

	Measured	PCA-MLR			PMF		
		Predicted	r^2^	%Error ^a^	Predicted	r^2^	%Error
PFBA	0.080	0.080	0.52	0	0.054	0.18	−33
PFPeA	0.043	0.043	0.81	0	0.035	0.60	−19
PFHxA	0.043	0.043	0.72	0	0.036	0.81	−16
PFHpA	0.028	0.028	0.75	0	0.021	0.65	−25
PFOA	0.202	0.202	0.72	0	0.168	0.68	−17
PFNA	0.084	0.084	0.55	0	0.069	0.71	−18
PFDA	0.098	0.098	0.77	0	0.080	0.77	−18
PFUnDA	0.155	0.155	0.81	0	0.139	0.83	−10
PFDoDA	0.033	0.033	0.91	0	0.026	0.89	−21
PFTrDA	0.060	0.060	0.82	0	0.045	0.79	−25
PFTeDA	0.009	0.009	0.70	0	0.007	0.63	−22
PFOS	0.165	0.165	0.45	0	0.190	0.63	15
∑_12_PFASs	0.999	0.999	0.98	0	0.982	0.99	−2

^a^%Error = (modeled concentration-measured concentration) × 100/measured concentration.
