# Supplementary material for: Spatial distribution and source apportionment of PFASs in surface sediments from five lake regions, China
Source: Sci Rep. 2016 Mar 7;6:22674. doi: 10.1038/srep22674 (PMC4780192; doi:10.1038/srep22674)
Supplement: Supplementary Information [file srep22674-s1.pdf]

Supporting information for

Spatial distribution and source apportionment of PFASs in surface sediments from five lake regions, China

Yanjie Qi [a, b], Shouliang Huo [a*], Beidou Xi [a], Shibin Hu [b], Jingtian Zhang [a], Zhuoshi He [a]

[a] State Key Laboratory of Environmental Criteria and Risk Assessment, Chinese Research Academy of Environmental Science, Beijing 100012, China

[b] College of Natural Resources and Environment, Northwest A&F University, Yangling 712100, China

[*]Corresponding author: Tel.: +86 (10) 84937970. Fax: +86 (10) 84913805. E-mail: huosl@craes.org.cn.

Number of tables: 3

Number of figures: 2

Number of pages: 8

Supporting information legends

Table S1 | Abbreviations, mass transitions, retention times (min) for the studied perfluoroalkyl substances (PFASs)

Table S2 | Geographic and limnological features of forty-eight lakes and two reservoirs studied

Table S3 | Limits of detection (ng/mL), limits of quantitation (ng/mL), blank spike recoveries (%, n=5) and matrix spike recoveries (%, n=5) for PFASs (n indicates the number of samples analyzed; mean ± SD) [a]

Figure S1 | A map plotting for forty-eight lakes and two reservoirs studied. (We created the map using the ArcGIS 9.2 software for windows 2000/XP/VISTA/7 and the Microsoft Office 2010 software for windows 2003/XP/VISTA/7.)

Figure S2 | Concentrations (ng/g dw) of PFASs in surface sediments from China, Japan, Austria, Germany, Australia, the USA and Canada (including sample size ≥1; No bar: no corresponding values) [8--15]. (We created the map using the Microsoft Office 2010 software for windows 2003/XP/VISTA/7.)

Table S1 | Abbreviations, mass transitions, retention times (min) for the studied perfluoroalkyl substances (PFASs)

Analyte	Acronym	Precursor/product ion	Retention time	Quantification by internal standard
**Target analytes**				
Perfluorobutyric acid	PFBA	212.9/168.9	1.68	$^{13}C_4$-PFBA
Perfluoropentanoic acid	PFPeA	262.9/218.9, 262.9/69.0	2.19	$^{13}C_4$-PFBA
Perfluorohexanoic acid	PFHxA	312.9/268.9, 312.9/119.1	2.78	$^{13}C_2$-PFHxA
Perfluoroheptanoic acid	PFHpA	362.9/318.9, 362.9/168.9	3.41	$^{13}C_2$-PFHxA
Perfluorooctanoate acid	PFOA	412.9/368.9, 412.9/169.0	4.00	$^{13}C_4$-PFOA
Perfluorononanoic acid	PFNA	462.9/418.9, 462.9/218.9	4.53	$^{13}C_5$-PFNA
Perfluorodecanoic acid	PFDA	512.9/468.9, 512.9/268.9	5.03	$^{13}C_2$-PFDA
Perfluoroundecanoic acid	PFUnDA	562.9/518.8, 562.9/268.8	5.48	$^{13}C_2$-PFUnDA
Perfluorododecanoic aicd	PFDoDA	612.9/568.9, 612.9/318.8	5.83	$^{13}C_2$-PFDoDA
Perfluorotridecanoic acid	PFTrDA	662.9/618.9, 662.9/368.9	6.15	$^{13}C_2$-PFDoDA
Perfluorotetradecanoic acid	PFTeDA	712.9/668.9, 712.9/368.9	6.42	$^{13}C_2$-PFDoDA
Perfluorohexadecanoic acid	PFHxDA	812.9/768.9, 812.9/468.9	6.85	$^{13}C_2$-PFDoDA
Perfluorooctadecanoic acid	PFOcDA	912.9/869.1, 912.9/568.9	7.22	$^{13}C_2$-PFDoDA
Perfluorobutane sulfonic acid	PFBS	298.9/99, 298.9/79.9	2.29	$^{18}O_2$-PFHxS
Perfluorohexane sulfonic acid	PFHxS	398.9/99, 398.8/79.9	3.44	$^{18}O_2$-PFHxS
Perfluorooctane sulfonic acid	PFOS	498.9/99, 498.9/79.9	4.56	$^{13}C_4$-PFOS
Perfluorodecane sulfonic acid	PFDS	598.9/99, 598.9/79.9	5.46	$^{13}C_4$-PFOS
**Internal standards**				
Perfluoro-n-[1,2,3,4-$^{13}C_4$] butanoic acid	$^{13}C_4$-PFBA	216.9/171.9	1.67	$^{13}C_8$-PFOA
Perfluoro-n-[1,2-$^{13}C_2$] hexanoic acid	$^{13}C_2$-PFHxA	314.9/269.9	2.77	$^{13}C_8$-PFOA
Perfluoro-n-[1,2,3,4-$^{13}C_4$] octanoic acid	$^{13}C_4$-PFOA	416.9/371.9	3.99	$^{13}C_8$-PFOA
Perfluoro-n-[1,2,3,4,5-$^{13}C_5$] nonannoic acid	$^{13}C_5$-PFNA	467.9/422.9	4.52	$^{13}C_8$-PFOA
Perfluoro-n-[1,2-$^{13}C_2$] decanoic acid	$^{13}C_2$-PFDA	514.9/469.9	5.02	$^{13}C_8$-PFOA
Perfluoro-n-[1,2-$^{13}C_2$] undecanoic acid	$^{13}C_2$-PFUnDA	564.9/519.8	5.46	$^{13}C_8$-PFOA
Perfluoro-n-[1,2-$^{13}C_2$] dodecanoic acid	$^{13}C_2$-PFDoDA	614.9/569.9	5.82	$^{13}C_8$-PFOA
Perfluoro-1-hexane [$^{18}O_2$] sulfonate	$^{18}O_2$-PFHxS	402.9/103.0	3.43	$^{13}C_8$-PFOS
Perfluoro-1- [1,2,3,4-$^{13}C_4$] octanesulfonate	$^{13}C_4$-PFOS	502.9/99.1	4.57	$^{13}C_8$-PFOS

Table S2 | Geographic and limnological features of forty-eight lakes and two reservoirs studied

Lake region	Lake	Serial number	Sampling time	Sample site	Position	Lake area (km^2)	Mean depth (m)	Catchment area (km^2)
East Plain Lake Region	Lake Wabu [1]	1	2012.04	Anhui Province	116°48´～117°01´E 32°23´～32°33´N	163	2.42	800
	Lake Longgan [1]	2	2012.06	Anhui Province	115°19´～116°17´E 29°52´～30°05´N	316	3.78	5,511
	Lake Shengjin [1]	3	2012.07	Anhui Province	116°58´～117°14´E 30°15´～30°28´N	78.5	1.26	1,554
	Lake Xiangjian [1]	4	2011.10	Anhui Province	117°15´～117°45´E 33°06´～33°12´N	45.0	0.93	-
	Lake Chengdong [1]	5	2012.04	Anhui Province	116°18´～116°28´E 32°12´～32°22´N	120	1.50	2,128
	Lake Chengxi [1]	6	2012.04	Anhui Province	116°01´～116°18´E 32°11´～32°33´N	199	2.70	1,750
	Lake Huayuan [1]	7	2011.10	Anhui Province	117°45´～117°53´E 32°55´～33°02´N	34.0	1.35	875
	Lake Baidang [1]	8	2012.07	Anhui Province	117°19´～117°27´E 30°47´～30°51´N	39.7	3.06	775
	Lake Bohu [1]	9	2012.06	Anhui Province	116°19´～116°33´E 30°04´～30°15´N	180	4.41	-
	Lake Wuchang [1]	10	2012.06	Anhui Province	116°36´～116°53´E 30°14´～30°20´N	101	3.43	1,084
	Lake Huangdahu [1]	11	2012.06	Anhui Province	116°14´～116°33´E 29°56´～30°08´N	299	3.94	-
	Lake Tuohu [1]	12	2011.10	Anhui Province	117°45´～117°51´E 33°09´～33°17´N	40.0	1.20	-
	Lake Nvshan [1]	13	2011.10	Anhui Province	117°58´～118°14´E 32°50´～33°02´N	105	1.71	4,215
	Lake Chaohu [1]	14	2011.12	Anhui Province	117°16´～117°05´E 31°25´～31°43´N	770	2.69	9,258
	Lake Luoma [1]	15	2013.06	Jiangsu Province	118°04´～118°18´E 34°00´～34°14´N	260	3.30	51,215
	Lake Cheng [1]	16	2013.07	Jiangsu Province	120°48´～120°52´E 31°10´～31°14´N	45.0	1.83	-
	Lake Yuandang [1]	17	2013.07	Jiangsu Province	120°51´～120°54´E 31°03´～31°05´N	12.9	1.38	-
	Lake Yaohu [1]	18	2013.08	Jiangsu Province	119°30´～119°40´E 31°30´～31°40´N	89.0	1.10	-
	Lake Gucheng [1]	19	2013.08	Jiangsu Province	118°53´～118°57´E 31°14´～31°18´N	24.5	1.56	248
	Lake Gehu [1]	20	2013.08	Jiangsu Province	119°44´～119°53´E 31°29´～31°42´N	147	1.19	-
	Lake Yangcheng [1]	21	2013.07	Jiangsu Province	120°39´～120°51´E 31°21´～31°30´N	119	1.40	-
	Lake Sanjiu [1]	22	2013.08	Jiangsu Province	119°48´E 31°22´N	23.2	1.85	-
	Lake Shijiu [1]	23	2013.08	Jiangsu Province	118°46´～118°56´E 31°23´～31°33´N	210	4.08	18,600
	Lake Gaoyou [1]	24	2013.06	Jiangsu Province	119°06´～119°25´E 32°42´～33°04´N	675	1.44	158,148
	Lake Nansi [1]	25	2013.06	Jiangsu Province	116°34´～117°21´E 34°27´～35°20´N	1,098	1.46	30,453
	Lake Shaobo [1]	26	2013.06	Jiangsu Province	119°23´～119°30´E 32°30´～32°40´N	77.0	1.10	-
	Lake Hongze [1]	27	2013.06	Jiangsu Province	118°10´～118°52´E 33°06´～33°40´N	1,577	1.77	156,000
	Lake Kuncheng [1]	28	2013.07	Jiangsu Province	120°43´～120°46´E 31°33´～31°36´N	18.0	1.78	-
	Lake Baima [1]	29	2013.06	Jiangsu Province	119°03´～119°11´E 33°09´～33°19´N	108	0.97	950
Mengxin Plateau Lake Region	Lake Bositeng [1]	30	2011.10	Xinjiang Uygur Autonomous Region	86°41´～87°27´E 41°49´～42°08´N	992	8.08	27,000
	Lake Wulungu [1]	31	2011.10	Xinjiang Uygur Autonomous Region	87°02´～87°35´E 47°02´～47°25´N	753	8.00	32,000
	Lake Jili [1]	32	2011.10	Xinjiang Uygur Autonomous Region	87°20´～87°32´E 46°51´～47°00´N	174	9.90	-
	Lake Hulun [1]	33	2011.10	Inner Mongolia Autonomous Region	116°58´～117°48´E 48°30´～49°20´N	2,339	5.92	37,214
	Lake Wuliangsu [1]	34	2011.10	Inner Mongolia Autonomous Region	108°43´～108°57´E 40°47´～41°03´N	233	1.12	11,800
Tibetan Plateau Lake Region	Lake Qinghai [1]	35	2012.07	Qinghai Province	99°36´～100°47´E 36°32´～37°15´N	4,340	17.9	29,661
	Lake Basongcuo [2]	36	2012.07	Tibet Autonomous Region	93°57´E 30°01´N	27.0	120 (Max.)	-
	Lake Yamdrok [1]	37	2012.07	Tibet Autonomous Region	90°21´～91°05´E 28°16´～29°11´N	638	20.0～40.0	5,422
	Lake Namucuo [1]	38	2012.07	Tibet Autonomous Region	90°16´～91°03´E 30°30´～30°56´N	1,962	-	8,649
Yungui Plateau Lake Region	Lake Yihai [3, 4]	39	2010.08	Sichuan Province	102°14´E 28°44´N	1.00	9.80	-
	Daqiao Reservoir [5]	40	2010.08	Sichuan Province	102°12´E 28°41´N	23.5	-	796
	Lake Qionghai [1]	41	2010.08	Sichuan Province	102°16´～102°21´E 27°47´～27°52´N	31.0	10.3	109
	Lake Mahu [1]	42	2010.08	Sichuan Province	103°47´～103°48´E 28°23´～28°26´N	7.30	65.7	97
	Lake Shudu [6]	43	2010.06	Yunnan Province	99°57´E 27°55´N	1.70	7.80 (Max.)	14
	Lake Qilu [1]	44	2010.07	Yunnan Province	102°43´～102°49´E 24°08´～24°13´N	36.9	4.03	341
	Lake Chenghai [1]	45	2010.06	Yunnan Province	100°38´～100°41´E 26°27´～26°38´N	77.2	25.7	229
	Lake Lugu [1]	46	2010.06	Yunnan Province	100°45´～100°50´E 27°41´～27°45´N	48.5	40.3	171
Northeast Plain Lake Region	Lake Wudalianchi [1]	47	2012.07	Heilongjiang Province	126°06´～126°15´E 48°40´～48°47´N	16.4	1.00-12.00	-
	Lake Xingkai [1]	48	2012.07	Heilongjiang Province	131°58´～132°51´E 44°32´～45°21´N	1,080	6.28	56,000
	Dahuofang Reservoir [7]	49	2012.07	Liaoning Province	120°20´～125°15´E 41°31´～42°15´N	22.0-91.2	-	5,437
	Lake Jingbo [1]	50	2012.07	Heilongjiang Province	128°37´～129°03´E 43°46´～44°03´N	91.5	12.9	11,820

Table S3 | Limits of detection (ng/mL), limits of quantitation (ng/mL), blank spike recoveries (%, n=5) and matrix spike recoveries (%, n=5) for PFASs (n indicates the number of samples analyzed; mean ± SD) [a]

Acronym	Limits of detection	Limits of quantitation	Blank spike recoveries	Matrix spike recoveries	
				2 ng/mL	20 ng/mL
**Target analytes**					
PFBA	0.01	0.05	104±5	88±9	96±7
PFPeA	0.01	0.03	101±4	87±12	95±9
PFHxA	0.01	0.04	97±3	92±8	104±6
PFHpA	0.02	0.06	102±5	109±9	98±7
PFOA	0.02	0.05	96±4	114±8	102±6
PFNA	0.02	0.06	112±5	109±10	97±7
PFDA	0.02	0.05	98±9	91±13	95±10
PFUnDA	0.02	0.08	102±6	111±10	115±8
PFDoDA	0.01	0.03	99±4	91±10	105±7
PFTrDA	0.01	0.05	96±7	88±12	108±9
PFTeDA	0.01	0.03	92±9	82±14	90±11
PFHxDA	0.06	0.20	89±8	77±13	84±11
PFOcDA	0.11	0.39	77±10	69±15	75±13
PFBS	0.02	0.06	98±3	90±7	102±8
PFHxS	0.01	0.04	99±4	109±9	93±6
PFOS	0.01	0.04	101±4	94±9	97±7
PFDS	0.01	0.03	96±5	92±8	90±8
**Internal standards**					
$^{13}C_4$-PFBA				93±9	
$^{13}C_2$-PFHxA				92±7	
$^{13}C_4$-PFOA				90±4	
$^{13}C_5$-PFNA				87±8	
$^{13}C_2$-PFDA				85±5	
$^{13}C_2$-PFUnDA				89±7	
$^{13}C_2$-PFDoDA				83±9	
$^{18}O_2$-PFHxS				90±7	
$^{13}C_4$-PFOS				92±6	

[a] The spike recoveries of PFASs were calculated using the equation: spike recovery = ($C_{sample + spiked}$ - $C_{sample)}$ / C_{spiked} × 100%, where $C_{sample + spiked}$ is the concentration of PFASs in a spiked sample, C_{sample} is the concentration of PFASs in the sample (same as above without spiked target compounds, C_{spiked} is the concentration of the spiked target compound. The recoveries of the stable isotope-labeled internal standards were calculated by the ratio of concentrations in the samples compared with those in the standard solutions.

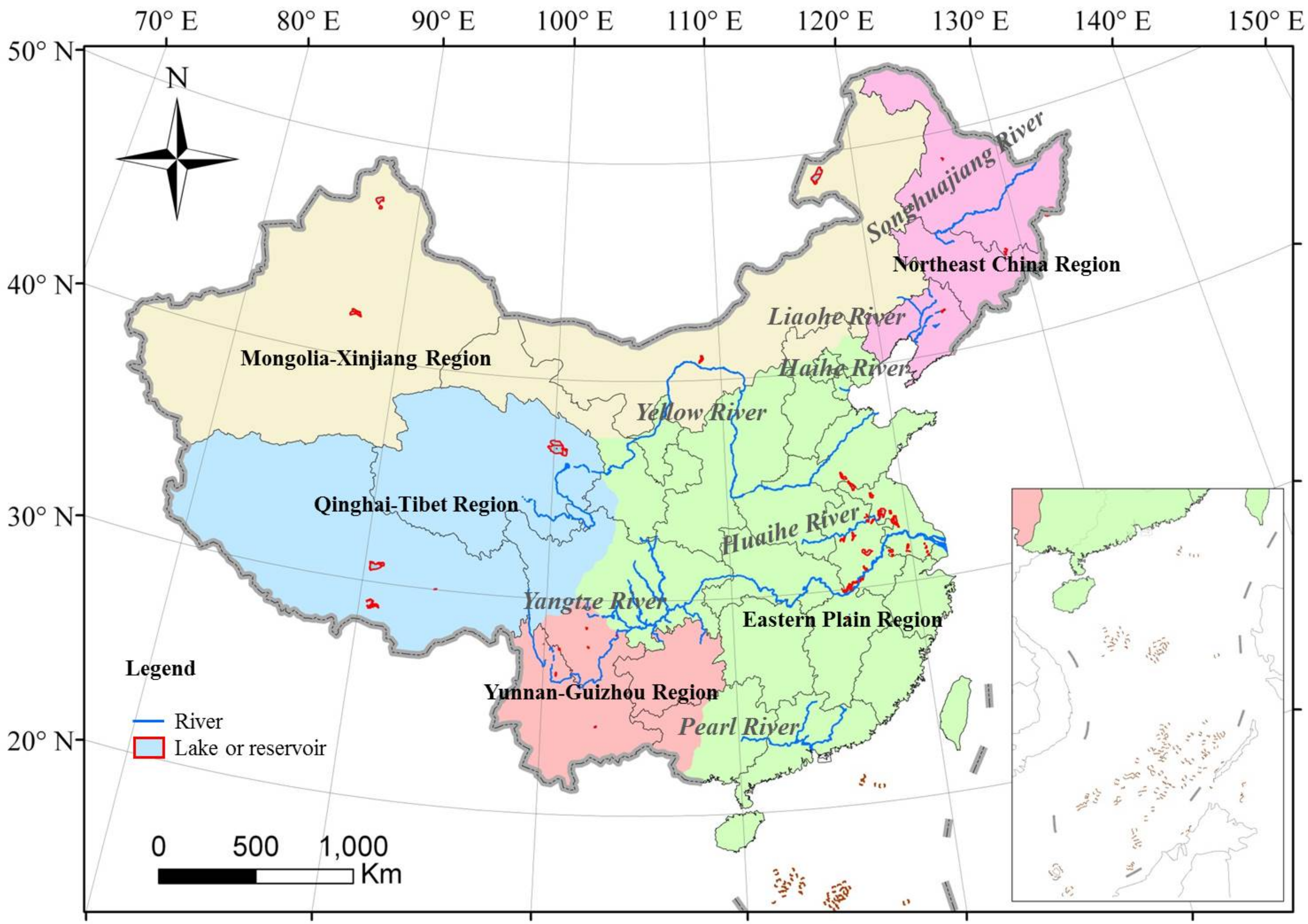

Figure S1 | A map plotting of forty-eight lakes and two reservoirs studied. (We created the map using the ArcGIS 9.2 software for windows 2000/XP/VISTA/7 and the Microsoft Office 2010 software for windows 2003/XP/VISTA/7.)

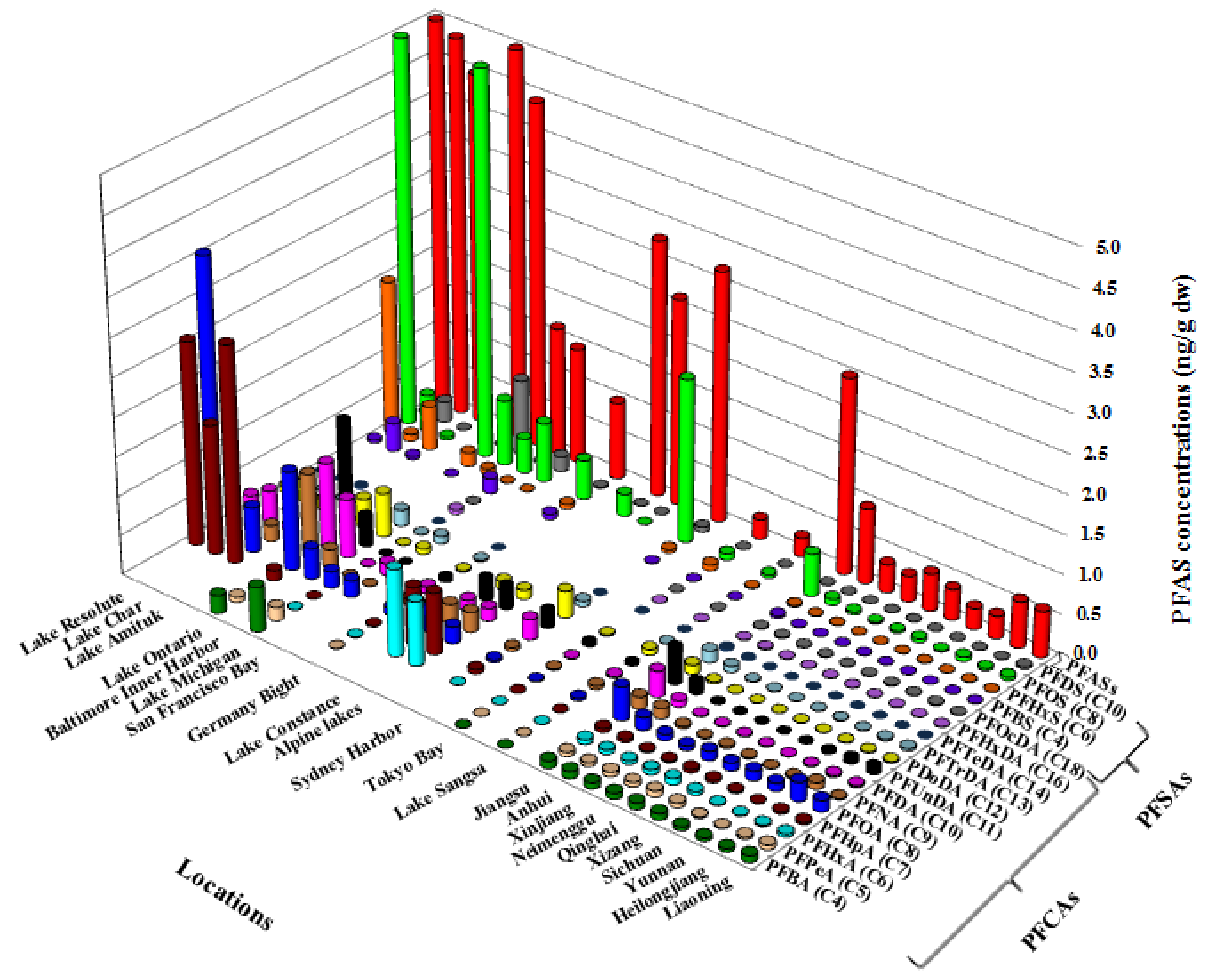

Figure S2 | Concentrations (ng/g dw) of PFASs in surface sediments from China, Japan, Austria, Germany, Australia, the USA and Canada (including sample size ≥1; No bar: no corresponding values) [8-15]. (We created the map using the Microsoft Office 2010 software for windows 2003/XP/VISTA/7.)

Literature Cited

1. Wang, S. M. & Dou, H. S. *Chinese lakes 1st edn* (Beijing: Science Press, 1998). (in Chinese)
2. *Pagsum Lake*. (2012) Available at: https://en.wikipedia.org/wiki/Lake_Cuogao. (Accessed: 24th December 2012)
3. Luo, Y., Xu, X. N. & Yan, W. Characteristics and evaluation of geological relic landscape in Yihai-Xiaoxiangling Mountains in Mianning country and Xide country. *Journal of Geological Hazards and Environment Preservation* **17**, 23-28 (2006). (in Chinese)
4. Yang, Y. T. & Liang, C. Research on runoff forecasting and optimal operation of Daqiao Reservoir in Xichang. *Sichuan Water Power* **27**, 77-80 (2008). (in Chinese)
5. *Yihai*. (2012) Available at: http://baike.baidu.com/link?url=-rc7C3SZKwP6Q_1t6KgSkYdaVhkUJa23LboIxmb2PfifN9YqQxMExXGhV5a4U 8I1lvrqAokA4LDuO9 sSk6SFRuoVgWm0EGXKf6Ll7HA7y7. (Accessed: 24th December 2012)
6. Jones, R. T. et al. Holocene environmental change at Lake Shudu, Yunnan Province, southwestern China. *Hydrobiologia* **693**, 223-235 (2012).
7. Pen, X. C., *Lake environment in China Vol. 3* (Beijing: Maritime Press, 1995). (in Chinese)
8. Ahrens, L. et al. Partitioning behavior of per- and polyfluoroalkyl compounds between pore water and sediment in two sediment cores from Tokyo Bay, Japan. *Environ. Sci. Technol.* **43**, 6969-6975 (2009).
9. Clara, M. et al. Perfluorinated alkylated substances in the aquatic environment: An Austrian case study. *Water Res.* **43**, 4760-4768 (2009).
10. Zhao, Z., Xie, Z. Y., Tang, J. H., Zhang, G. & Ebinghaus, R. Spatial distribution of perfluoroalkyl acids in surface sediments of the German Bight, North Sea. *Sci. Total Environ.* **511**, 145-152 (2015).
11. Thompson, J. et al. Perfluorinated alkyl acids in water, sediment and wildlife from Sydney Harbour and surroundings. *Mar. Pollut. Bull.* **62**, 2869-2875 (2011).
12. Yeung, L. W. Y. et al. Perfluoroalkyl substances and extractable organic fluorine in surface sediments and cores from Lake Ontario. *Environ. Int.* **59**, 389-397 (2013).
13. Higgins, C. P., Field, J. A., Criddle, C. S. & Luthy, R. G. Quantitative determination of perfluorochemicals in sediments and domestic sludge. *Environ. Sci. Technol.* **39**, 3946-3956 (2005).
14. Codling, G. et al. Historical trends of inorganic and organic fluorine in sediments of Lake Michigan. *Chemosphere* **114**, 203-209 (2014).
15. Stock, N. L., Furdui, V. I., Muir, D. C. G. & Mabury, S. A. Perfluoroalkyl contaminants in the Canadian Arctic: Evidence of atmospheric transport and local contamination. *Environ. Sci. Technol.* **41**, 3529-3536 (2007).
